# Evidence from household surveys for measuring coverage of newborn care practices

**DOI:** 10.7189/jogh.07.020503

**Published:** 2017-12

**Authors:** Deborah Sitrin, Jamie Perin, Lara ME Vaz, Liliana Carvajal–Aguirre, Shane M Khan, Joy Fishel, Agbessi Amouzou

**Affiliations:** 1 Saving Newborn Lives, Save the Children, Washington, D.C., USA; 2 Institute for International Programs, Johns Hopkins School of Public Health, Baltimore, Maryland, USA; 3 Data and Analytics, Division of Data, Research and Policy, UNICEF, New York, New York, USA; 4 ICF, Rockville, Maryland, USA

## Abstract

**Background:**

Aside from breastfeeding, there are little data on use of essential newborn care practices, such as thermal protection and hygienic cord care, in high mortality countries. These practices have not typically been measured in national household surveys, often the main source for coverage data in these settings. The *Every Newborn* Action Plan proposed early breastfeeding as a tracer for essential newborn care due to data availability and evidence for the benefits of breastfeeding. In the past decade, a few national surveys have added questions on other practices, presenting an opportunity to assess the performance of early breastfeeding initiation as a tracer indicator.

**Methods:**

We identified twelve national surveys between 2005–2014 that included at least one indicator for immediate newborn care in addition to breastfeeding. Because question wording and reference populations varied, we standardized data to the extent possible to estimate coverage of newborn care practices, accounting for strata and multistage survey design. We assessed early breastfeeding as a tracer by: 1) examining associations with other indicators using Pearson correlations; and 2) stratifying by early breastfeeding to determine differences in coverage of other practices for initiators vs non–initiators in each survey, then pooling across surveys for a meta–analysis, using the inverse standard error as the weight for each observation.

**Findings:**

Associations between pairs of coverage indicators are generally weak, including those with breastfeeding. The exception is drying and wrapping, which have the strongest association of any two interventions in all five surveys where measured; estimated correlations for this range from 0.47 in Bangladesh’s 2007 DHS to 0.83 in Nepal’s 2006 DHS. The contrast in coverage for other practices by early breastfeeding is generally small; the greatest absolute difference was 6.7%, between coverage of immediate drying for newborns breastfed early compared to those who were not.

**Conclusions:**

Early initiation of breastfeeding is not a high performing tracer indicator for essential newborn care practices measured in previous national surveys. To have informative data on whether newborns are getting life–saving services, standardized questions about specific practices, in addition to breastfeeding initiation, need to be added to surveys.

Every year, 2.7 million babies die during the first month of life, largely from preventable causes [1]. The World Health Organization has prioritized several newborn care practices that could be used at home or facility to prevent many of these unnecessary deaths – thermal care to prevent hypothermia, hygienic cord and skin care to prevent infections, and early and exclusive breastfeeding [2]. Strong evidence on the mortality impact of specific practices is mostly unavailable, but the benefits are likely substantial. Delphi–based expert panels suggested clean postnatal practices could reduce deaths due to infections by 40% [3] and thermal care could reduce deaths due to preterm complications by 20% [4]. More robust evidence exists for the impact of early and exclusive breastfeeding, with a recent cohort trial finding late breastfeeding initiators had higher neonatal mortality (41% if initiated 2–24 hours after birth, 79% if more than 24 hours after birth) and infant mortality, which persisted even in exclusively breastfed babies, suggesting both early and exclusive breastfeeding independently reduce mortality [5].

Despite the importance of these behaviors, most countries do not have coverage data to know if they are practiced. Very few national health information systems collect data on these practices [6] and national surveys, such as the Demographic and Health Survey (DHS) and the Multiple Indicator Cluster Survey (MICS), usually only include questions on breastfeeding, but not other newborn care practices [7]. The *Every Newborn* Action Plan’s (ENAP) Measurement Improvement Roadmap [8] was an important step forward in building momentum for improving measurement on newborn care, though mainly focused on coverage indicators for interventions to manage small or sick newborns, such as Kangaroo Mother Care and infection management. ENAP proposed early breastfeeding as a tracer for essential newborn care—preventive and supportive care all newborns need—due to the strong evidence for breastfeeding and its availability from DHS and MICS. However, the correlation between breastfeeding and use of other newborn care practices has not been examined, so it is not known if breastfeeding coverage corresponds with coverage of other practices. In the absence of data, it is generally assumed coverage of these practices is low in settings with high neonatal mortality. For example, a recent effort to model the impact of improving coverage of various interventions in high–burden countries assumed baseline coverage of clean postnatal practices and simple thermal were each just 11%, while coverage of exclusive breastfeeding at one month was estimated to be 62% (early initiation was not included) [4]. The lack of coverage data for other practices makes it difficult to monitor the effectiveness of strategies to promote them or identify unreached populations [9].

Population–based household surveys, particularly DHS and MICS, are often the main source for intervention coverage data in low– and middle–income countries. In many of these countries, a large proportion of deliveries occur outside health facilities and routine data systems are often weak [10]. Surveys are also used to collect sociodemographic data to identify inequities [11,12]. DHS and MICS measure contacts with the health system during the antenatal, birth, and postnatal periods, but contacts alone are poor indications of the content and quality of care and should not be used as a stand–in for effective coverage of high impact interventions [13,14]. Of the essential newborn care practices, only breastfeeding questions were included in standard DHS and MICS questionnaires until 2016. However, surveys are adapted to each country and a few national surveys prior to 2016 included additional questions on newborn care, presenting an opportunity to assess the performance of breastfeeding initiation as a tracer for essential newborn care practices.

This study first examines how DHS and MICS from 2005–2014 have asked about newborn care practices and standardizes the calculation of indicators, to the extent possible, to examine and compare coverage levels across countries. We then investigate the utility of early initiation of breastfeeding as a tracer indicator for essential newborn care. This analysis is especially important as countries weigh the need to include additional questions on newborn care into their next national survey. While both DHS and MICS recently included additional standardized newborn indicators in their model questionnaires based on global consensus around indicators that could be collected in household surveys (**Table 1**), most questions are optional and countries must choose to include them [15,16].

**Table 1:** New questions in DHS (Phase 7) and MICS6 related to newborn care practices

**DHS Women’s Model Questionnaire:**
434 Immediately after the birth, was (NAME) put on your chest?
434A Was (NAME)’s bare skin touching your bare skin?
**DHS Optional Newborn Module:**
NB1 Was (NAME) wiped dry within a few minutes after birth?
NB2 How long after the birth was (NAME) bathed for the first time?
NB3 CHECK PLACE OF DELIVERY
NB4 What was used to cut the cord? (non–institutional births only)
NB5 Was it new or had it ever been used before? (non–institutional births only)
NB5A Was it boiled before it was used to cut the cord? (non–institutional births only)
NB6 Was anything applied to the stump of the cord at any time?
NB7 What was applied?
CH1 CHECK SUBSTANCES APPLIED TO CORD
CH2 Was chlorhexidine applied to the stump at any time?
CH3 How long after the cord was cut was chlorhexidine fist applied?
CH4 For how many days was chlorhexidine applied to the stump?
CH5 How many times per day was chlorhexidine applied to the stump: once a day, twice a day, three times a day, or four or more times a day?
**MICS6 Questionnaire for Individual Women:**
**MN23** Immediately after the birth, was (***name***) put directly on the bare skin of your chest? [WITH PHOTO OF SKIN–TO–SKIN POSITION]
**MN24** Before being placed on the bare skin of your chest, was the baby wrapped up?
**MN25** Was (***name***) dried or wiped soon after birth?
**MN26** How long after the birth was (***name***) bathed for the first time?
*Recommended only for countries with high NMR, large programs on cord care, large proportion of non–facility births:*
**MN27** Check MN20: Was the child delivered in a health facility?
**MN28** What was used to cut the cord? (non–institutional births only)
**MN29** Was the instrument used to cut the cord boiled or sterilised prior to use? (non–institutional births only)
**MN30** After the cord was cut and until it fell off, was anything applied to the cord?
**MN31** What was applied to the cord?

## METHODS

We reviewed publicly available DHS and MICS reports from 2005–2014 to identify surveys capturing newborn care practices in addition to breastfeeding. Once surveys were identified, analysis proceeded with two primary components, the first descriptive and the second to examine relationships between indicators of coverage. Twelve national surveys in eight countries (four in South or Southeast Asia, three in sub–Saharan Africa, one in western Asia) were found that measured at least one indicator for immediate newborn care in addition to initiation of breastfeeding. Three countries (Bangladesh, Nepal, and Armenia) had more than one survey and therefore the potential to compare coverage over time. **Table 2** briefly describes all twelve surveys by the number of births recorded in the two years prior to each survey, the proportion of births occurring in non–institutional settings, and the proportion of Caesarean births. We compared how questions were asked in different surveys based on the following criteria: 1) wording of questionnaire items, 2) how responses were quantified, 3) target population of interest (eg, facility or home births), 4) reference period (eg, in the two or three years preceding survey), and 5) birth subset (all births in reference period or only most recent birth).

**Table 2:** Twelve nationally representative household surveys that included measures of essential newborn care beyond breastfeeding

Country	Year	Type	Number of Households Surveyed	Number of births in past two years	Number (%) of non–institutional births in past two years	Number (%) of cesarean births in the past two years
Armenia	2005	DHS	4022	621	8 (1%)	59(10%)
Armenia	2010	DHS	3535	675	1 (0%)	90(13%)
Bangladesh	2007	DHS	8583	2469	1949 (79%)	262(11%)
Bangladesh	2011	DHS	14068	3483	2337 (67%)	648(19%)
Bangladesh	2014	DHS	14228	3283	1932 (59%)	805(25%)
Ghana	2014	DHS	6062	2517	698 (28%)	282(11%)
India	2005	DHS	76010	20837	9585 (46%)	2438(12%)
Malawi	2014	MICS	20772	7576	563 (7%)	412(5%)
Nepal	2006	DHS	6672	2270	1817(80%)	58(3%)
Nepal	2011	DHS	7874	2103	1156(55%)	127(6%)
Nigeria	2013	DHS	23364	13570	8345(61%)	326(2%)
Timor–Leste	2009	DHS	7516	4006	3107(78%)	74(2%)

DHS – Demographic and Health Survey, MICS – Multiple Indicator Cluster Survey

We estimated coverage of newborn care practices as defined by each survey, and then, to the extent possible, standardized indicators across surveys. Our standardized indicators are defined in **Table 3**, which also shows the comparability of these definitions to data that will be collected with the new DHS and MICS questionnaires. Given differences in wording and answer options, indicator numerators could not be perfectly harmonized across surveys. For example, the timing of interventions was recorded as an exact amount in some surveys, and as timing relative to other events in other surveys.

**Table 3:** Standardized definitions of newborn coverage indicators used for this analysis and comparability to DHS (Phase 7) module and MICS6

Indicator group	Standardized definition	Comparability to DHS (Phase 7)	Comparability to MICS6
Breastfeeding initiation	Put to breast within one hour of birth	Comparable	Comparable
Thermal care	Dried within five minutes of birth OR before delivery of the placenta	Somewhat comparable (DHS does not reference exact time or delivery of placenta' to 'DHS does not use five minutes or delivery of placenta for time reference)	Somewhat comparable (MICS6 does not reference exact time or delivery of placenta' to 'MICS6 does not use five minutes or delivery of placenta for time reference)
	Wrapped within five minutes of birth OR before delivery of the placenta	Not comparable (not collected in DHS)	Not comparable (MICS6 asks if baby wrapped up before placed on mother’s bare chest.)
	Neonate put on the belly or breast before delivery of the placenta OR directly on the bare skin of your chest	Somewhat comparable (DHS specifies bare skin must be touching in 2 questions)	Somewhat comparable (MICS specifies bare skin must be touching in 2 questions and a photo)
	Not given a bath in the first 24 h after birth	Comparable	Comparable
Hygienic cord care	A new or sterilized (boiled) instrument was used to cut the umbilical cord, or a clean delivery kit was used	Somewhat comparable (DHS does not ask about clean delivery kit)	Somewhat comparable (MICS6does not ask about clean delivery kit)
	No substance was applied to the umbilical cord after it was cut	Comparable	Comparable

DHS – Demographic and Health Survey, MICS – Multiple Indicator Cluster Survey

We standardized indicator denominators by recall period and population, using the shortest reference period across surveys (last birth in the two years preceding survey), and the smallest common reference population (births that were delivered at home). We used these standard populations and definitions to estimate coverage, accounting for strata and the multistage survey design in each case [17,18]. Once we standardized these coverage estimates, we examined their levels across countries and across time for multiple surveys in a single country.

We then examined the associations between various coverage indicators among those surveyed to determine how well early breastfeeding functions as a tracer for other indicators of newborn care. We used Pearson correlations to describe associations between each pair of estimated coverages [19]. We expected *a priori* that some types of coverage would be positively correlated: that is, an infant receiving a specific intervention would be likely to receive a related intervention (for example, an infant who is dried may often be wrapped as well). We also hypothesized some coverages would be negatively correlated, indicating that an infant would be less likely to receive an intervention if another intervention had been received (for example, wrapping and placing skin–to–skin). We examined relationships with breastfeeding within one hour of delivery for each indicator at the individual level with these correlations.

We also aimed to describe the coverage of newborn care practices among newborns breastfed early and examine if it differed from coverage among newborns who did not breastfeed early. For each survey, we stratified by breastfeeding within one hour, and compared coverage estimates for each group. We statistically determined the difference between the coverage of newborn care practices for these groups. We then pooled observed differences across surveys in a meta–analysis. In order that different surveys contribute to the estimate overall, we used the inverse standard error as the weight for each observation. Using inverse standard errors as weights allows survey estimates with more uncertainty to contribute less information to overall estimates, per standard meta–analysis protocol. [20].

## RESULTS

Two surveys (Nepal 2011 and Nigeria 2013) measured all seven indicators considered for the second part of our analysis (**Table 4**). Surveys in Armenia and Ghana each only collected one indicator of interest other than breastfeeding. The India 2005 survey included multiple newborn care practices, but asked as a composite question so it is not possible to tease out coverage of each practice. Except for early breastfeeding, coverage for other practices was often measured only for home births. However, in Armenia, Ghana, Malawi, and Bangladesh (2007 and 2014), some items were measured for all non–Caesarean births. Questions and response categories for each survey can be found in Table S1 in **Online Supplementary Document(Online Supplementary Document)** and respective coverage estimates from official survey reports in Figure S1 in **Online Supplementary Document(Online Supplementary Document)**, though coverage is generally not comparable across surveys due to question wording, their reference populations, and time periods. We excluded four surveys from further analysis given the limited amount of comparable indicators – Armenia (2005 and 2010), Ghana, and India – leaving eight surveys for standardized coverage measurement.

**Table 4:** Immediate newborn care indicators included for each of twelve recent surveys

	Armenia	Armenia	Ghana	India	Malawi	Bangladesh			Nepal		Nigeria	Timor–Leste
**Indicator**	**2005**	**2010**	**2014**	**2005**	**2014**	**2007**	**2011**	**2014**	**2006**	**2011**	**2013**	**2014**
Breastfed within first hour	A	A	A	A	A	A	A	A	A	A	A	A
Dried				H	H	H	A	H	H	H	H	H
Wrapped			A	H		H	A		H	H	H	
Bathed after 24 h				H	H	H	H	A	H	H	H	H
New or boiled blade				H	H	H	H	H	H	H	H	H
Nothing applied to cord					H	H	H	A	H	H	H	H
Skin to skin or baby put on mother’s belly or chest	A	A						A		H	H	

A – Surveys that collected data for all non– Caesarean last births, H – Surveys that collected data only for home births

**Figure 1:**
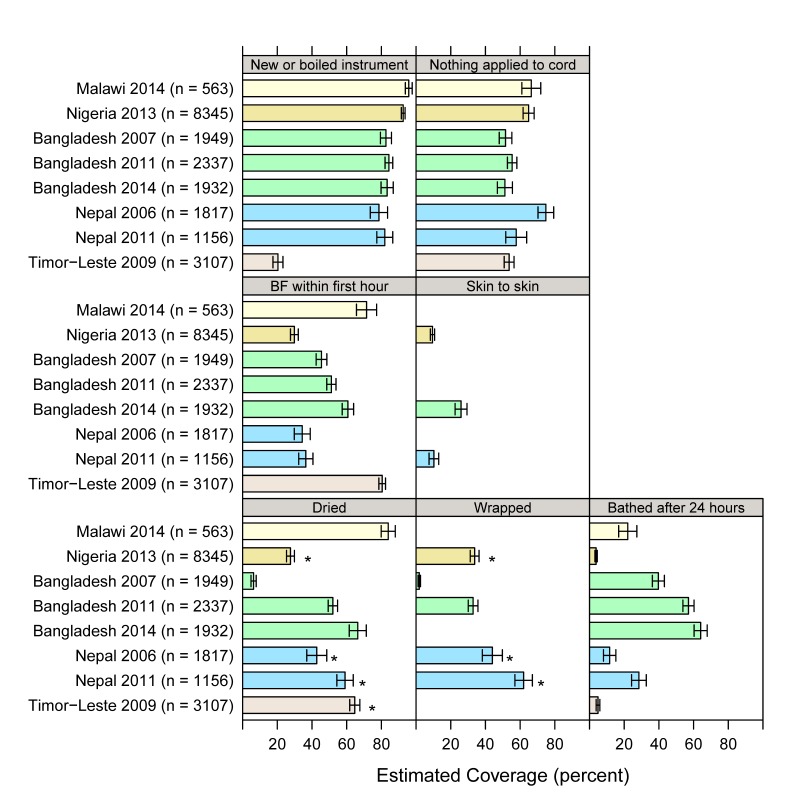
Standardized coverage estimates for eight national surveys, with 95% confidence limits, for most recent births that were delivered in non–institutional settings in the two years preceding survey. Asterisk indicates that “before placenta delivery” was used for time reference, as opposed to “within five minutes” for drying or wrapping.

We used the standardized definitions for most recent births which were also in non–institutional settings in the two years preceding the survey. Resulting coverage estimates are shown with 95% confidence intervals in **Figure 1** and Table S2 in **Online Supplementary Document(Online Supplementary Document)**. Using a new or boiled instrument to cut the umbilical cord is generally the highest estimated coverage, except in Timor–Leste. Placing the baby skin–to–skin or on the mother’s belly or chest was measured in only three of the eight surveys and generally had the lowest estimated coverage among the newborn care practices. Some measures had wide variation across surveys, such as drying, which ranges from 6.3% in Bangladesh (2007) to 84% in Malawi (2014). Trends in coverage over time can also be inferred from **Figure 1** for the two countries with more than one survey. Drying, wrapping, and delayed bathing increased in both Bangladesh and Nepal. There is no apparent change over time in using a new or boiled instrument in either country. Nepal had no change in early breastfeeding, but Bangladesh had a small increase between 2007 and 2014. For no application to the cord, there is a decline of 17 percentage points (95% confidence interval 6.8–28.0) in Nepal from 2006 to 2011. The 2011 survey added a follow–up question on what was applied to the cord with chlorhexidine as an answer option. Yet chlorhexidine use fails to explain this decrease in dry cord care: changing the 2011 coverage indicator to include nothing *or* chlorhexidine applied resulted in little change in the 2011 coverage estimate (only 1% respond regarding chlorhexidine, so coverage changes from 57.7% to 58.7%). Coverage for dry cord care was unchanged over time in Bangladesh.

**Figure 2:**
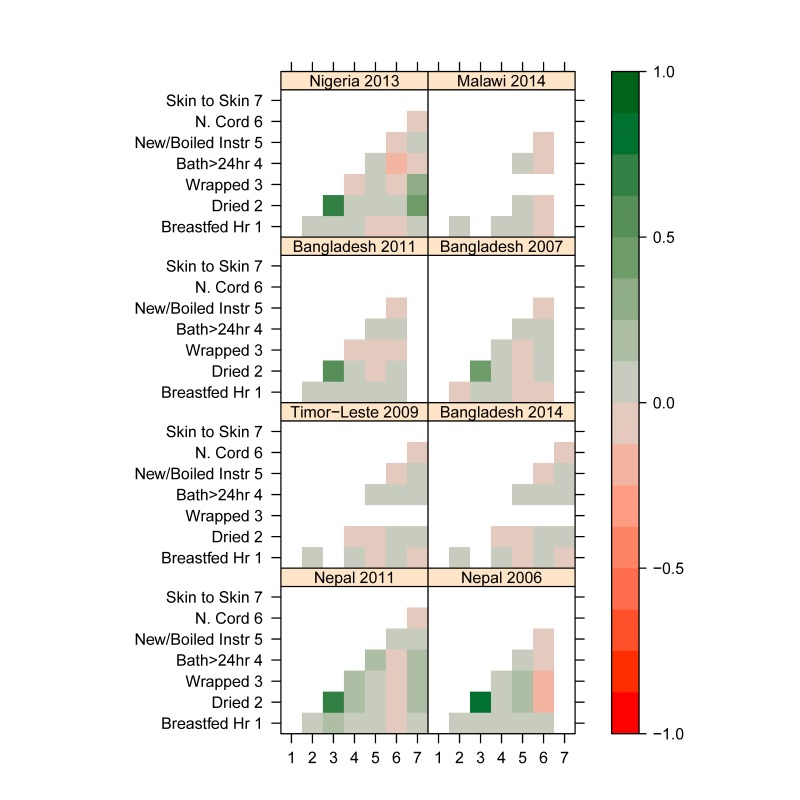
Correlation matrices for eight national surveys and seven standardized coverage indicators, for most recent births in the two years preceding survey that were delivered in non–institutional settings. Strong correlations are indicated by dark green, negative correlations are indicated by red.

We used these standardized coverage estimates in each survey to examine the associations between different newborn care practices at the individual level, to see whether neonates who receive a specific intervention are likely to receive another. We estimated the Pearson correlation between coverage indicators for each survey for all available measurements. The resulting associations are shown in **Figure 2** as a map, where interventions that tend to occur together are darker green the more they are positively correlated, and interventions that tend to occur separately are darker red the more they are negatively correlated. (See Table S3 in Online **Supplementary Document** for correlations.) Associations between pairs of newborn coverage indicators are generally weak, including those with breastfeeding. The exception is for drying and wrapping, which have the strongest association of any two interventions in all five surveys where they were measured, with an estimated correlation of 0.65 in Nigeria 2013; 0.83 and 0.73 in Nepal 2006 and 2001, respectively; and 0.47 and 0.58 in Bangladesh 2007 and 2011. Other correlations above 0.2 are between being placed “skin–to–skin” (includes babies placed mother’s belly or chest in Nigeria and Nepal, babies placed on the mother’s bare skin in Bangladesh) and both drying and wrapping in Nigeria 2013 and Nepal 2011. In Nigeria, infants placed “skin–to–skin” are somewhat more likely to have been dried (correlation 0.42) and wrapped (correlation 0.35). In Nepal 2011, infants placed “skin–to–skin” are also more likely to have been dried (correlation 0.24) and wrapped (correlation 0.23). Skin–to–skin and drying were not related in Bangladesh 2014 (correlation 0.013), while wrapping was not asked.

In addition to these associations, we stratified surveys by early breastfeeding and examined the differences in coverage for those with early breastfeeding compared to those who did not breastfeed early. The estimated contrasts in coverage are shown for each survey and pooled across surveys in **Table 5**. The contrast in coverage between neonates by early breastfeeding is generally small, with pooled differences less than seven percentage points for each indicator. The absolute difference in coverage of essential practices between newborns breastfed early and those who were not ranged from less than one percent for having nothing applied to the cord to a difference of 6.7% for drying.

**Table 5:** Newborn coverage indicators, by survey and early breastfeeding status. The differences in coverage between those with early breastfeeding and those without was meta–analyzed for the pooled difference across surveys

		Among those breastfed in first hour	Among those not breastfed in first hour	Difference (BF – not BF)	95% Confidence interval (%)	
**Description**	**Survey**	**Estimate (%)**	**Estimate (%)**	**Estimate (%)**	**Lower**	**Upper**
**Bathed after 24 h**	Malawi 2014	21.6	23.1	–1.5	–12.8	9.9
	Nigeria 2013	4.9	3.2	1.6	0.3	2.9
	Bangladesh 2007	43.3	36.4	6.9	1.3	12.5
	Bangladesh 2011	61.5	52.0	9.5	4.7	14.3
	Bangladesh 2014	66.7	59.0	7.8	1.3	14.3
	Nepal 2006	12.2	10.6	1.6	–2.0	5.3
	Nepal 2011	36.2	24.8	11.3	3.9	18.8
	Timor–Leste 2009	4.6	6.3	–1.7	–4.0	0.6
	**Pooled**			**4.0**	**1.1**	**6.9**
**Dried**	Malawi 2014	85.9	79.2	6.7	–3.0	16.4
	Nigeria 2013*	30.3	26.7	3.6	0.1	7.1
	Bangladesh 2007	5.2	7.4	–2.2	–4.6	0.1
	Bangladesh 2011	55.0	48.9	6.2	1.3	11.1
	Bangladesh 2014	69.5	61.8	7.7	2.4	13.0
	Nepal 2006*	49.5	39.5	10.0	3.1	16.9
	Nepal 2011*	64.3	56.0	8.3	1.7	15.0
	Timor–Leste 2009*	67.6	51.5	16.1	10.0	22.1
	**Pooled**			**6.7**	**2.2**	**11.2**
**New or boiled blade**	Malawi 2014	96.0	95.4	0.6	–4.0	5.2
	Nigeria 2013	92.5	92.6	–0.1	–2.0	1.9
	Bangladesh 2007	82.5	82.7	–0.2	–4.2	3.8
	Bangladesh 2011	85.2	83.5	1.7	–2.0	5.5
	Bangladesh 2014	82.7	84.7	–1.9	–6.0	2.1
	Nepal 2006	82.0	76.9	5.1	–0.1	10.4
	Nepal 2011	85.7	80.2	5.5	0.1	11.0
	Timor–Leste	21.0	17.2	3.8	–0.4	8.1
	**Pooled**			**1.3**	**–0.4**	**3.0**
**Nothing applied to cord**	Malawi 2014	66.2	66.9	–0.7	–10.9	9.5
	Nigeria 2013	60.0	66.7	–6.7	–10.7	–2.8
	Bangladesh 2007	48.0	54.2	–6.1	–11.9	–0.4
	Bangladesh 2011	57.9	52.4	5.5	1.0	10.0
	Bangladesh 2014	49.6	52.7	–3.1	–9.9	3.6
	Nepal 2006	78.3	73.4	4.9	–0.1	9.8
	Nepal 2011	62.7	55.1	7.6	0.3	14.9
	Timor–Leste	52.3	60.2	–7.9	–13.7	–2.1
	**Pooled**			**–0.1**	**–5.5**	**3.7**
**Skin to skin**	Nigeria 2013	12.4	8.4	4.1	1.6	6.5
	Bangladesh 2014	25.9	25.8	0.1	–5.1	5.3
	Nepal 2011	13.1	8.7	4.4	0.5	8.4
	**Pooled**			**3.6**	**1.7**	**5.5**
**Wrapped**	Bangladesh 2007	1.7	2.1	–0.4	–1.6	0.8
	Bangladesh 2011	32.6	32.9	–0.3	–4.8	4.2
	Nepal 2006†	50.0	41.1	8.9	1.8	15.9
	Nepal 2011†	71.2	56.4	14.8	7.4	22.1
	Nigeria 201†	37.7	32.2	5.6	1.7	9.5
	**Pooled**			**4.9**	**0.1**	**9.6**

*Dried before delivery of placenta.

†Wrapped before delivery of placenta.

## DISCUSSION

With little guidance on how to measure care for newborns and, until recently, little global attention on newborn health, few nationally representative household surveys have measured newborn care practices. In a ten year period, we identified only twelve surveys across eight countries, and several of these surveys asked about few practices. There was inconsistency across surveys in how and to whom newborn care questions were asked, which limits comparability. DHS and MICS have now offered standard questions to improve the consistency of data in coming years.

Early initiation of breastfeeding does not appear to be a high performing tracer indicator for essential newborn care, since it is poorly correlated with the all the other elements of newborn care in this analysis. Nor was there much difference in coverage of other practices when comparing babies who were breastfed within an hour and those who were not. In fact, no single practice was a good predictor for the coverage of other practices. In particular, there was very little correlation between coverage of any thermal care practices and coverage of cord care (and some had negative associations). Only drying and wrapping were highly correlated. Wrapping was not added to the DHS questionnaire because overlap between these two practices was previously seen in program surveys and thus it was deemed unnecessary to collect both [7]. Wrapping and “skin–to–skin” contact also appear to be weakly correlated in Nigeria 2013 and Nepal 2011. However, true skin–to–skin care and wrapping may be mutually exclusive events since a baby that is wrapped will not have exposed skin to place against the mother’s bare skin. The correlation found in these two surveys may be explained by the fact that the question (Was the baby placed on the mother’s belly/breast before delivery of the placenta?) did not specify skin–to–skin exposure, unlike how it was asked in Bangladesh 2014 or will be asked in future DHS and MICS (as shown in **Table 2**).

Indicator validation studies in Mozambique, Kenya, and Mexico have shown mothers have difficulty accurately reporting newborn care practices, though findings were inconsistent with drying, breastfeeding within an hour, and skin–to–skin contact meeting validation criteria in at least one study but not in other studies [21–24]. The weak correlations found in this study could be due to invalidity of indicators. On the other hand, newborn care practices may simply be inconsistently applied, which could also explain why correlations are weak with the exception of drying and wrapping. These past validation studies also had some design limitations. They could not include home birth observations, while this study was limited to only analyzing home births. Since validation for home births presents feasibility and ethical problems, triangulation of intervention coverage data with outcomes for babies born at home could be used to assess the plausibility of coverage levels. In addition, the validation studies did not ask all questions the same way they are asked in the new DHS and MICS questionnaires (including the question on initiation of breastfeeding) and did not examine the umbilical cord care practices now measured in DHS and MICS. While recall bias is a flaw of household surveys, most countries have no other means to assess coverage of these life–saving interventions. To have informative data on whether newborns are getting the services they need, questions about specific practices, aside from breastfeeding initiation, need to be added to surveys.

Nonetheless, surveys can mitigate bias due to mothers not witnessing certain practices, understanding terminology or what they saw being done for their baby, or remembering what was done, especially if a long time has passed since their last birth [25,26]. New DHS and MICS questions were developed and field tested to improve reporting. For example, validation research found a two–item question sequence improved mothers’ reporting of skin–to–skin care, resulting in DHS and MICS adding two questions to their new questionnaires [22]. MICS6 also included a photo of a baby in the skin–to–skin position to help mothers understand the question. Mothers have also been shown to have difficulty reporting the exact timing of practices [16], so DHS and MICS limit the number of practices for which the mother is asked to give timing (breastfeeding initiation and first bathing) and simplified the need to recall precise timing by not requiring recall in minutes for practices within the first hour after birth. Instrument sterilization in facilities likely occurs outside the delivery room and many mothers who delivered in a facility report not knowing if the instrument was clean when asked [7], so DHS and MICS only ask questions on cutting the umbilical cord to mothers who delivered at home. Standard probes and follow–on questions could further improve recall and reduce use of leading questions [26,27], though not yet part of DHS or MICS interviewer manuals [28,29].

Many of the surveys reviewed in this paper only asked questions on newborn care for home births. The new DHS and MICS questionnaires are now designed to ask most questions for both facility and home births, because omitting facility births creates an information gap, especially as facility delivery rates rise; the exception is on questions on the instrument used to cut the cord [15,16]. In the future, therefore, correlations between newborn care practices for facility births can be examined. At the same time, routine data systems should be strengthened to capture newborn care practices delivered at facility and triangulate with survey data, as well as collect data on services to treat rare complications that cannot be reliably collected through national surveys.

After standardizing indicators to the extent possible, we found reported use of a clean instrument for cutting the cord among non–institutional births was high (around 80% or more) in all countries except Timor–Leste, and remained high over consecutive surveys in Bangladesh and Nepal. Coverage of dry cord care was more moderate, with a decline in Nepal from 75% in 2006 to 58% in 2011. Changing the indicator to include chlorhexidine application could not explain the decline; coverage of chlorhexidine application was low because Nepal only decided to proceed with national implementation of chlorhexidine in late 2011 [30]. As countries adopt the 2013 WHO guidelines recommending chlorhexidine application for newborns born at home in settings with high neonatal mortality [31], the appropriate indicator will be nothing *or* chlorhexidine only applied to the cord stump. Given the interest this new intervention, countries will need to know coverage. Increasing awareness of chlorhexidine may also help reporting accuracy.

Coverage levels for clean cutting and dry cord care practices in the surveys analyzed in this paper are much higher than Bhutta et al’s modelled coverage estimate for the general category ‘clean postnatal care practices’, which was just 11% [4]. Bhutta’s definition included handwashing and skin cleansing and did not include hygienic cord care, which likely explains why coverage is so different than what we found in these surveys. At the same time, countries that ask questions about hygienic cord care in national surveys may be more invested in changing these practices, so coverage may be higher than would be found others. The same may not be true for other hygienic postnatal care practices, which may be closer to Bhutta’s estimate.

Early breastfeeding was generally moderate (ranging from 30% to 82%) with little change between surveys in Bangladesh and Nepal. These findings were in line with the average across all 75 countries tracked by *Countdown to 2015*, which was 50% [32]. Use of thermal care practices varied across countries, with drying, wrapping, and delayed bathing improving over time in Bangladesh and Nepal. Placing the neonate on the mother’s belly or breast or on the mother’s bare skin was low (10–25%). Overall, coverage estimates for thermal care practices in these surveys are substantially higher than Bhutta et al’s modelled coverage estimate for ‘simple thermal care’ (11%). Though again, Bhutta’s definition was not the same as used in this paper.

As the global community makes new commitments to the health and survival of newborns through the Sustainable Development Goals [33], countries need to know how newborns are cared for, beyond whether they are breastfed early. This study found coverage can vary greatly for different practices as well as differences across countries. There may not be a single way forward to improve the care of newborns, so country level data on multiple newborn care practices are critical. Essential newborn care may have even greater benefit for preterm babies, so having data to guide efforts to improve coverage of all practices will be important to reducing child mortality, now that prematurity is the leading cause of death for children under 5 [1]. New standards in household surveys will increase the availability of coverage estimates for these life–saving interventions for a key vulnerable population.
