# Supplementary material for: Evidence from household surveys for measuring coverage of newborn care practices
Source: J Glob Health. 2017 Dec 20;7(2):020503. doi: 10.7189/jogh.07.020503 (PMC5804503; doi:10.7189/jogh.07.020503)

Online Supplementary Document

Sitrin et al. Evidence from household surveys for estimating coverage of newborn care practices

J Glob Health 2017;7:020503

Table S1. **Questionnaire items in twelve household surveys related to newborn care practices**

Table S2. **Standardized coverage estimates for eight nationally representative household surveys with measures of essential newborn care. Coverage estimates are shown for births in non-institutional settings in the two years preceding survey.**

Table S3. **Correlations among standardized coverage estimates of essential newborn care, for most recent births in non-institutional settings in the two years preceding survey.**

Figure S1. **Reported coverage in eleven household surveys that measured practices of newborn care.**

**Table S1. Questionnaire items in twelve household surveys related to newborn care practices**	India DHS 2005/6	Armenia DHS 2005	Armenia DHS 2010	Bangladesh DHS 2007	Bangladesh DHS 2011	Bangladesh DHS 2014	Nepal DHS 2006	Nepal DHS 2011	Nigeria DHS 2013	Ghana DHS 2014	Malawi MICS 2014	Timor-Leste DHS 2009
Drying	447B. Was the baby immediately wiped dry then wrapped without being bathed? *(Y/N/DK, home births)* *(Y/N/DK, home births)*	-	-	430G. How long after (NAME) was born was the body wipes (dried)? *(mins, home births in past 3yrs)*	435I. How long after delivery was (NAME) dried? *(Categorical, normal deliveries in past 3yrs)*	435AH. How long after delivery was (NAME) dried? *(Categorical, normal deliveries in past 3yrs)*	432D. Was (NAME) dried before the placenta was delivered? *(Y/N/DK, home births)*	431F. Was (NAME) dried before the placenta was delivered? *(Y/N/DK, home births)*	437F. Was (NAME) dried before the placenta was delivered? *(Y/N/DK, home births)*	-	MN20A. Was (*name*) dried or wiped after delivery? *(Y/N/DK, all births)*<br><br>MN20B. How soon after birth was (*name*) dried or wiped? *(immed/hrs, all births)*	443D Was (NAME) dried before the placenta was delivered? *(Y/N/DK, home births)*
Wrapping	See above	-	-	430H. How long after (NAME) was born was the body wrapped? *(mins, home births in past 3yrs)*	435J. How long after delivery was (NAME) wrapped? *(Categorical, normal deliveries in past 3yrs)*		432E. Was (NAME) wrapped in cloth before the placenta was delivered? *(Y/N/DK, home births*	431H. Was (NAME) wrapped in cloth before the placenta was delivered? *(Y/N/DK, home births)*	437H. Was (NAME) wrapped in cloth before the placenta was delivered? *(Y/N/DK, home births)*	441A How long after birth was (NAME) wrapped? *(Categorical, all births)*	-	-

Bathing	See above	-	-	430J. How long after delivery was (NAME) bathed for the first time? *(hrs/days/wks, home births in past 3yrs)*	435H. How long after delivery was (NAME) bathed for the first time? *(hrs/days/wks, normal deliveries in past 3yrs)*	435AG How long after delivery was (NAME) bathed for the first time?	432F. How long after delivery was (NAME) bathed for the first time? *(hrs/days/wks, home births)*	431I. How long after delivery was (NAME) bathed for the first time? *(hrs/days/wks, home births)*	437I. How long after delivery was (NAME) bathed for the first time? *(hrs/days/wks, home births)*	441 How long after birth was (NAME) bathed for the first time? *(Categorical, all births)*	MN20C. How soon after birth was (*name*) bathed for the first time? *(Immed/hrs, all births)*	443E How long after birth was (NAME) bathed for the first time? *(Categorical, home births)*
Skin-to-skin	-	455A After the birth, was (NAME) put directly on the bare skin of your chest? *(Y/N/DK, all births)*	433A Immediately after the birth, was (NAME) put directly on the bare skin of your chest? *(Y/N/DK, all births)*	-	-	443AI After the birth, was (NAME) put directly on the bare skin of your chest?	-	431G. Was (NAME) placed on your belly/breast before delivery of the placenta? *(Y/N/DK, home births)*	437G. Was (NAME) placed on your belly/breast before delivery of the placenta? *(Y/N/DK, home births)*	-	-	-
Cord cutting	447c. Was a clean blade used to cut the cord? *(Y/N/DK, home births)*	-	-	430B. What was used to cut the cord? *(Categorical, home births in past 2yrs)*<br>430C. Was the __ (INSTRUMENT) boiled before the cord was cut? *(Y/N/DK, home births in past 3yrs)*	435D. What was used to cut the cord? *(Categorical, home births)*<br>435E. Was the __ (INSTRUMENT IN 435D) boiled before the cord was cut? *(Y/N/DK, home births in past 3yrs)*	435AC What was used to cut the cord?<br>435AD Was the (INSTRUMENT IN 435AC) boiled before the cord was cut?	432B. When (NAME) was born, what instrument was used to cut the umbilical cord? *(Categorical, home births)*	431C. When (NAME) was born, what instrument was used to cut the umbilical cord? *(Categorical, home births)*	437C. When (NAME) was born, what instrument was used to cut the umbilical cord? *(Categorical, home births)*	-	MN18A. What was used to cut the cord of (*name*)? *(Categorical, home births)*<br>MN18B. Was the instrument used to cut the cord of (*name*) boiled prior to use? *(Y/N/DK, home births)*	443A When (NAME) was born, what instrument was used to cut the umbilical cord? *(Categorical, home births)*

Material put on cord	-	-	-	430D. Was anything applied to the cord immediately after cutting and tying it? *(Y/N/DK, home births in past 2yrs)*<br><br>430E. What was applied to the cord after it was cut and tied? *(Categorical, home births in past 3yrs)*	435F. Was anything applied to the cord immediately after cutting and tying it? *(Y/N/DK, all normal deliveries)*<br><br>435G. What was applied to the cord after it was cut and tied? *(Categorical, normal deliveries in past 3yrs)*	435AE Was anything applied to the cord immediately after cutting and tying it?	432C. Was anything placed on the stump after the umbilical cord was cut? *(Y/N/DK, home births)*	431D. Was anything placed on the stump after the umbilical cord was cut? *(Y/N/DK, home births)*<br><br>431E. What was placed on the stump? *(Categorical, home births)*	437D. Was anything applied on the stump after the umbilical cord was cut? *(Y/N/DK, home births)*<br><br>437E. What was applied on the stump? *(Categorical, home births)*	-	MN18C. Was anything applied to the cord of (*name*) after the cord was cut and tied until the cord fell off? *(Y/N/DK, home births)*<br><br>MN18D. What was applied to the cord? *(Categorical, home births*	443B Was anything placed on the stump after the umbilical cord was cut? *(Y/N/DK, home births)*<br><br>443C. What was placed on the stump? *(Categorical, home births)*
Clean Delivery Kit	447a. Was a disposable delivery kit used? *(Y/N/DK, home births)*	-	-	-	435C. Was a Clean Delivery Kit used during delivery of (NAME)? *(Y/N/DK, home births in past 3yrs)*	435AB Was a Clean Delivery Kit used during the delivery of (NAME)?	432A. Was a special clean delivery kit used? *(Y/N/DK, home births)*	431B. Was a special clean delivery kit used? *(Y/N/DK, home births)*	437B. Was a special clean delivery kit used? *(Y/N/DK, home births)*	-	-	-
Breast-feeding initiation	467. How long after birth did you first put (NAME) to the breast? *(Immed/hrs/days, all births)*	457. How long after birth did you first put (NAME) to the breast? *(Immed/hrs/days, all births)*	455. How long after birth did you first put (NAME) to the breast? *(Immed/hrs/days, all births)*	449. How long after birth did you first put (NAME) to the breast? *(Immed/hrs/days, all births)*	453. How long after birth did you first put (NAME) to the breast? *(Immed/hrs/days, all births)*	455 How long after birth did you first put (NAME) to the breast?	450. How long after birth did you first put (NAME) to the breast? *(Immed/hrs/days, all births)*	449. How long after birth did you first put (NAME) to the breast? *(Immed/hrs/days, all births)*	455. How long after birth did you first put (NAME) to the breast? *(Immed/hrs/days, all births)*	455. How long after birth did you first put (NAME) to the breast? *(Immed/hrs/days, all births)*	MN25. How long after birth did you first put (NAME) to the breast? *(Immed/hrs/days, all births)*	461 How long after birth did you first put (NAME) to the breast? *(Immed/hrs/days, all births)*
Colostrum	-	-	-	449A Was (NAME) given colostrum immediately after his/her birth? *(Y/N/DK, all births)*	-	-	-	-	-	-	-	-

Table S2. Standardized coverage estimates for eight nationally representative household surveys with multiple measures of essential newborn care. Coverage estimates are shown for births in non-institutional settings in the two years preceding survey.

Description	N	Survey	Standardized Estimate	95% Confidence Lower	95% Confidence Upper
Bathed after 24 hours	563	Malawi 2014	22.1%	16.8%	27.3%
	13750	Nigeria 2013	3.8%	3.1%	4.5%
	1949	Bangladesh 2007	39.7%	36.2%	43.2%
	2337	Bangladesh 2011	57.0%	53.8%	60.3%
	1932	Bangladesh 2014	53.4%	50.7%	56.1%
	2770	Nepal 2006	11.6%	8.1%	15.2%
	2103	Nepal 2011	28.5%	24.2%	32.7%
	3107	Timor-Leste 2009	5.0%	4.0%	6.0%
Breastfed within first hour	563	Malawi 2014	71.5%	65.6%	77.3%
	13750	Nigeria 2013	29.8%	27.6%	32.1%
	1949	Bangladesh 2007	45.5%	42.4%	48.6%
	2337	Bangladesh 2011	51.2%	48.5%	53.8%
	1932	Bangladesh 2014	52.0%	49.6%	54.3%
	2770	Nepal 2006	34.4%	29.8%	39.0%
	2103	Nepal 2011	36.4%	32.3%	40.5%
	3107	Timor-Leste 2009	81.7%	80.3%	83.1%
Dried	563	Malawi 2014	84.0%	79.9%	88.0%
	13750	Nigeria 2013*	27.6%	25.3%	29.9%
	1949	Bangladesh 2007	6.3%	4.8%	7.8%
	2337	Bangladesh 2011	52.0%	49.3%	54.7%
	1932	Bangladesh 2014	67.0%	62.4%	71.6%
	2770	Nepal 2006*	42.8%	36.9%	48.6%
	2103	Nepal 2011*	59.1%	54.3%	63.8%
	3107	Timor-Leste 2009*	65.0%	62.4%	67.5%
New or boiled instrument	563	Malawi 2014	95.8%	93.8%	97.8%
	13750	Nigeria 2013	92.6%	91.5%	93.7%
	1949	Bangladesh 2007	82.6%	79.5%	85.8%
	2337	Bangladesh 2011	84.3%	82.1%	86.6%
	1932	Bangladesh 2014	84.3%	81.7%	86.8%
	2770	Nepal 2006	78.6%	73.6%	83.6%
	2103	Nepal 2011	82.0%	77.4%	86.6%
	3107	Timor-Leste 2009	20.0%	17.4%	22.7%
Nothing applied to cord	563	Malawi 2014	66.4%	61.0%	71.9%
	13750	Nigeria 2013	64.9%	61.7%	68.1%
	1949	Bangladesh 2007	51.6%	48.0%	55.3%
	2337	Bangladesh 2011	55.4%	52.7%	58.1%
	1932	Bangladesh 2014	50.6%	46.5%	54.8%
	2770	Nepal 2006	75.1%	70.7%	79.6%
	2103	Nepal 2011	57.7%	51.7%	63.8%
	3107	Timor-Leste 2009	53.3%	50.6%	55.9%
Skin to skin	13750	Nigeria 2013	9.5%	8.3%	10.7%
	1932	Bangladesh 2014	25.2%	22.0%	28.5%
	2103	Nepal 2011	10.3%	7.5%	13.1%

Wrapped	13750	Nigeria 2013**	33.8%	31.2%	36.3%
	1949	Bangladesh 2007	1.9%	1.2%	2.6%
	2337	Bangladesh 2011	32.9%	30.1%	35.7%
	2770	Nepal 2006**	44.2%	38.4%	50.0%
	2103	Nepal 2011**	62.0%	57.0%	67.1%

***** Dried before delivery of the placenta

****** Wrapped before delivery of the placenta

Table S3. Correlations among standardized coverage estimates of essential newborn care, for most recent births in non-institutional settings in the two years preceding survey.

Country	Year	Coverages		Correlation Estimate	95% CI Low	95% CI High	*p*
Bangladesh	2007	Breastfed within first hour	Dried	-0.030	-0.076	0.015	0.187
		Breastfed within first hour	Wrapped	0.000	-0.045	0.046	0.983
		Breastfed within first hour	Bathed after 24 hours	0.068	0.023	0.113	0.003
		Breastfed within first hour	New or boiled instrument	-0.016	-0.061	0.030	0.499
		Breastfed within first hour	Nothing applied to cord	-0.048	-0.093	-0.002	0.040
		Dried	Wrapped	0.471	0.435	0.506	<0.001
		Dried	Bathed after 24 hours	0.028	-0.017	0.073	0.226
		Dried	New or boiled instrument	-0.013	-0.058	0.032	0.567
		Dried	Nothing applied to cord	0.027	-0.019	0.072	0.248
		Wrapped	Bathed after 24 hours	0.068	0.023	0.113	0.003
		Wrapped	New or boiled instrument	-0.053	-0.098	-0.007	0.022
		Wrapped	Nothing applied to cord	0.002	-0.043	0.047	0.939
		Bathed after 24 hours	New or boiled instrument	0.062	0.017	0.107	0.007
		Bathed after 24 hours	Nothing applied to cord	0.024	-0.021	0.070	0.293
		New or boiled instrument	Nothing applied to cord	-0.030	-0.075	0.016	0.200
Bangladesh	2011	Breastfed within first hour	Dried	0.073	0.032	0.114	<0.001
		Breastfed within first hour	Wrapped	0.004	-0.037	0.046	0.833
		Breastfed within first hour	Bathed after 24 hours	0.070	0.028	0.111	0.001
		Breastfed within first hour	New or boiled instrument	0.024	-0.017	0.066	0.249
		Breastfed within first hour	Nothing applied to cord	0.057	0.015	0.098	0.007
		Dried	Wrapped	0.578	0.550	0.605	<0.001
		Dried	Bathed after 24 hours	0.046	0.005	0.086	0.029
		Dried	New or boiled instrument	-0.019	-0.060	0.022	0.368
		Dried	Nothing applied to cord	0.020	-0.021	0.061	0.349
		Wrapped	Bathed after 24 hours	-0.014	-0.055	0.027	0.508
		Wrapped	New or boiled instrument	-0.021	-0.062	0.021	0.328
		Wrapped	Nothing applied to cord	-0.004	-0.045	0.037	0.844
		Bathed after 24 hours	New or boiled instrument	0.081	0.040	0.122	<0.001
		Bathed after 24 hours	Nothing applied to cord	0.031	-0.011	0.071	0.145
		New or boiled instrument	Nothing applied to cord	-0.008	-0.049	0.033	0.702
Bangladesh	2014	Breastfed within first hour	Dried	0.063	0.017	0.109	0.007
		Breastfed within first hour	Bathed after 24 hours	0.053	0.007	0.098	0.024
		Breastfed within first hour	Clean instrument	-0.035	-0.081	0.011	0.134
		Breastfed within first hour	Nothing on cord	0.007	-0.040	0.054	0.761
		Breastfed within first hour	Skin to skin	-0.004	-0.050	0.042	0.857
		Dried	Bathed after 24 hours	-0.004	-0.049	0.041	0.863
		Dried	New or boiled instrument	-0.007	-0.052	0.038	0.746
		Dried	Nothing applied to cord	0.013	-0.032	0.059	0.568
		Dried	Skin to skin	0.013	-0.031	0.058	0.555
		Bathed after 24 hours	New or boiled instrument	0.027	-0.018	0.072	0.241
		Bathed after 24 hours	Nothing applied to cord	0.009	-0.037	0.054	0.710
		Bathed after 24 hours	Skin to skin	0.029	-0.016	0.074	0.202
		New or boiled instrument	Nothing applied to cord	-0.021	-0.066	0.025	0.381
		New or boiled instrument	Skin to skin	0.057	0.012	0.102	0.012
		Nothing applied to cord	Skin to skin	-0.045	-0.091	0.000	0.051

Table S3 (continued). Correlations among standardized coverage estimates of essential newborn care, for most recent births in non-institutional settings in the two years preceding survey.

				Correlation			
					95% CI		
Country	Year	Coverages		Estimate	Low	High	*p*
Nepal	2006	Breastfed within first hour	Dried	0.066	0.019	0.113	0.006
		Breastfed within first hour	Wrapped	0.058	0.011	0.105	0.016
		Breastfed within first hour	Bathed after 24 hours	0.014	-0.033	0.062	0.547
		Breastfed within first hour	New or boiled instrument	0.070	0.023	0.117	0.004
		Breastfed within first hour	Nothing applied to cord	0.012	-0.036	0.059	0.624
		Dried	Wrapped	0.826	0.811	0.841	<0.001
		Dried	Bathed after 24 hours	0.117	0.071	0.163	<0.001
		Dried	New or boiled instrument	0.151	0.104	0.196	<0.001
		Dried	Nothing applied to cord	-0.152	-0.198	-0.106	<0.001
		Wrapped	Bathed after 24 hours	0.092	0.045	0.138	<0.001
		Wrapped	New or boiled instrument	0.131	0.085	0.177	<0.001
		Wrapped	Nothing applied to cord	-0.131	-0.177	-0.084	<0.001
		Bathed after 24 hours	New or boiled instrument	0.108	0.061	0.154	<0.001
		Bathed after 24 hours	Nothing applied to cord	-0.095	-0.142	-0.048	<0.001
		New or boiled instrument	Nothing applied to cord	-0.113	-0.159	-0.066	<0.001
Nepal	2011	Breastfed within first hour	Dried	0.117	0.058	0.175	<0.001
		Breastfed within first hour	Wrapped	0.172	0.114	0.228	<0.001
		Breastfed within first hour	Bathed after 24 hours	0.116	0.057	0.174	<0.001
		Breastfed within first hour	New or boiled instrument	0.058	-0.001	0.117	0.055
		Breastfed within first hour	Nothing applied to cord	0.051	-0.009	0.110	0.096
		Breastfed within first hour	Skin to skin	0.078	0.019	0.136	0.010
		Dried	Wrapped	0.731	0.703	0.758	<0.001
		Dried	Bathed after 24 hours	0.138	0.080	0.195	<0.001
		Dried	New or boiled instrument	0.085	0.027	0.143	0.004
		Dried	Nothing applied to cord	-0.070	-0.129	-0.011	0.021
		Dried	Skin to skin	0.235	0.179	0.290	<0.001
		Wrapped	Bathed after 24 hours	0.133	0.075	0.190	<0.001
		Wrapped	New or boiled instrument	0.054	-0.004	0.113	0.070
		Wrapped	Nothing applied to cord	-0.071	-0.130	-0.011	0.019
		Wrapped	Skin to skin	0.227	0.171	0.282	<0.001
		Bathed after 24 hours	New or boiled instrument	0.176	0.119	0.233	<0.001
		Bathed after 24 hours	Nothing applied to cord	0.008	-0.052	0.067	0.803
		Bathed after 24 hours	Skin to skin	0.141	0.083	0.198	<0.001
		New or boiled instrument	Nothing applied to cord	0.019	-0.040	0.078	0.526
		New or boiled instrument	Skin to skin	0.112	0.054	0.170	<0.001
		Nothing applied to cord	Skin to skin	-0.099	-0.158	-0.040	0.001
Timor-Leste	2009	Breastfed within first hour	Dried	0.132	0.096	0.168	<0.001
		Breastfed within first hour	Bathed after 24 hours	-0.036	-0.072	0.001	0.057
		Breastfed within first hour	New or boiled instrument	0.025	-0.011	0.062	0.176
		Breastfed within first hour	Nothing applied to cord	-0.078	-0.114	-0.041	<0.001
		Dried	Bathed after 24 hours	0.008	-0.028	0.044	0.671
		Dried	New or boiled instrument	0.113	0.077	0.149	<0.001
		Dried	Nothing applied to cord	-0.052	-0.088	-0.016	0.005
		Bathed after 24 hours	New or boiled instrument	-0.019	-0.056	0.017	0.299
		Bathed after 24 hours	Nothing applied to cord	-0.022	-0.058	0.014	0.234
		New or boiled instrument	Nothing applied to cord	0.119	0.083	0.155	<0.001

Table S3 (continued). Correlations among standardized coverage estimates of essential newborn care, for most recent births in non-institutional settings in the two years preceding survey.

				Correlation			
					95% CI		
**Country**	**Year**	**Coverages**		**Estimate**	**Low**	**High**	***p***
Nigeria	2013	Breastfed within first hour	Dried	0.030	0.008	0.052	0.009
		Breastfed within first hour	Wrapped	0.066	0.044	0.088	<0.001
		Breastfed within first hour	Bathed after 24 hours	0.017	-0.005	0.039	0.123
		Breastfed within first hour	New or boiled instrument	-0.003	-0.025	0.019	0.779
		Breastfed within first hour	Nothing applied to cord	-0.086	-0.107	-0.064	<0.001
		Breastfed within first hour	Skin to skin	0.053	0.031	0.075	<0.001
		Dried	Wrapped	0.654	0.641	0.667	<0.001
		Dried	Bathed after 24 hours	0.006	-0.016	0.028	0.576
		Dried	New or boiled instrument	0.055	0.033	0.076	<0.001
		Dried	Nothing applied to cord	0.000	-0.022	0.022	0.982
		Dried	Skin to skin	0.420	0.401	0.437	<0.001
		Wrapped	Bathed after 24 hours	-0.008	-0.030	0.014	0.486
		Wrapped	New or boiled instrument	0.053	0.031	0.075	<0.001
		Wrapped	Nothing applied to cord	-0.013	-0.035	0.009	0.244
		Wrapped	Skin to skin	0.351	0.332	0.370	<0.001
		Bathed after 24 hours	New or boiled instrument	0.019	-0.003	0.041	0.083
		Bathed after 24 hours	Nothing applied to cord	-0.146	-0.168	-0.125	<0.001
		Bathed after 24 hours	Skin to skin	-0.031	-0.053	-0.010	0.005
		New or boiled instrument	Nothing applied to cord	-0.094	-0.116	-0.072	<0.001
		New or boiled instrument	Skin to skin	0.050	0.029	0.072	<0.001
		Nothing applied to cord	Skin to skin	-0.001	-0.023	0.021	0.902
Malawi	2014	Dried	Bathed after 24 hours	NE*			
		Dried	Nothing applied to cord	-0.047	-0.129	0.036	0.264
		Bathed after 24 hours	Nothing applied to cord	-0.006	-0.096	0.084	0.899

*Not estimable

Figure S1. Reported coverage in nine household surveys that measured practices of newborn care.

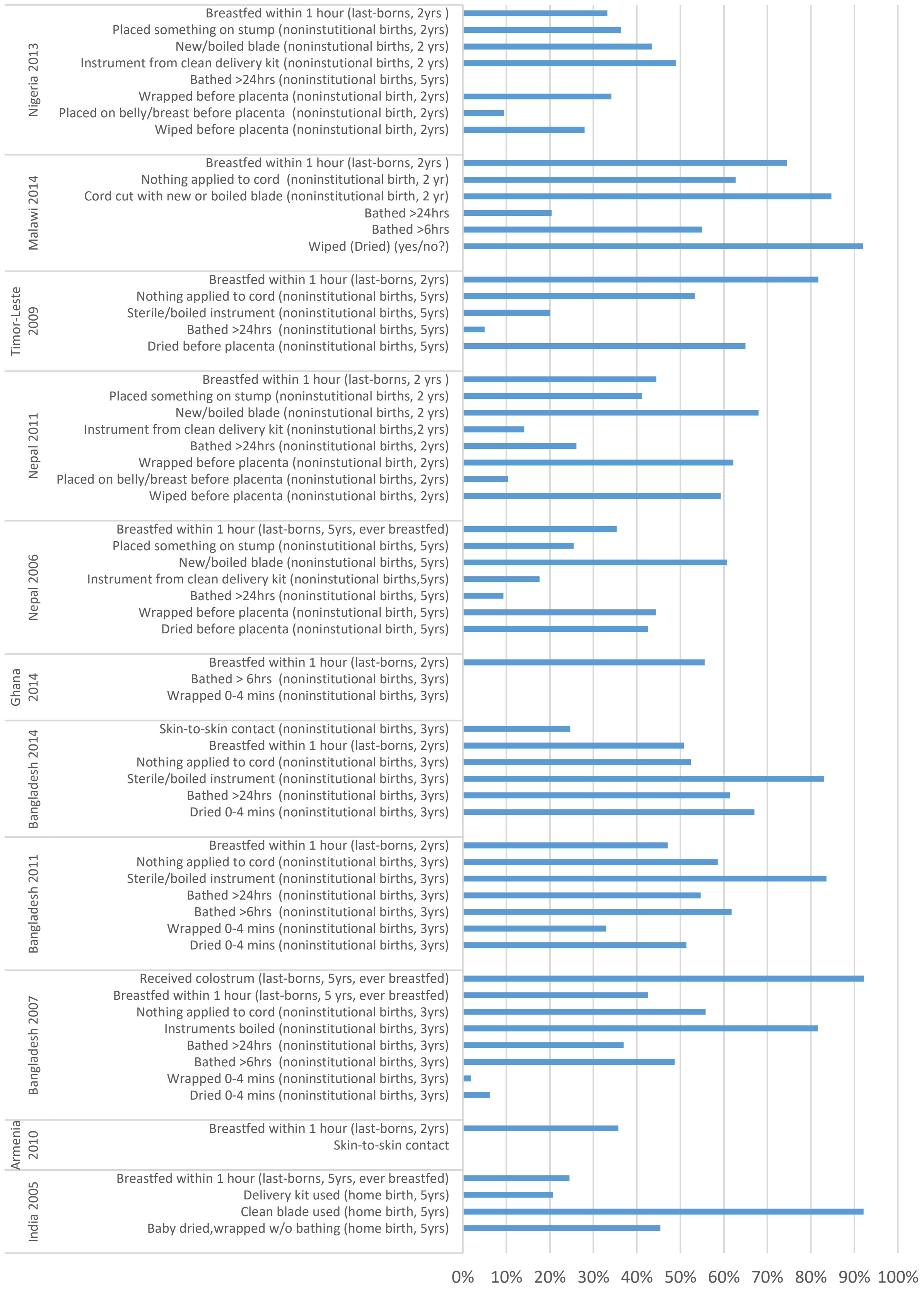

Supplement: Online Supplementary Document [file jogh-07-020503-s001.pdf]
